# Recapitulation of Fibromatosis Nodule by Multipotential Stem Cells in Immunodeficient Mice

**DOI:** 10.1371/journal.pone.0024050

**Published:** 2011-08-25

**Authors:** Jung-Pan Wang, Yun-Ju Hui, Shih-Tien Wang, Hsiang-Hsuan Michael Yu, Yi-Chao Huang, En-Rung Chiang, Chien-Lin Liu, Tain-Hsiung Chen, Shih-Chieh Hung

**Affiliations:** 1 Institute of Clinical Medicine, School of Medicine, National Yang-Ming University, Taipei, Taiwan; 2 Department of Pharmacology, School of Medicine, National Yang-Ming University, Taipei, Taiwan; 3 Department of Surgery, School of Medicine, National Yang-Ming University, Taipei, Taiwan; 4 Department of Orthopaedics and Traumatology, Taipei Veterans General Hospital, Taipei, Taiwan; 5 Medical Research and Education, Taipei Veterans General Hospital, Taipei, Taiwan; 6 Department of Radiation Oncology, H. Lee Moffitt Cancer Center and Research Institute, Tampa, Florida, United States of America; University of California, Merced, United States of America

## Abstract

Musculoskeletal fibromatosis remains a disease of unknown etiology. Surgical excision is the standard of care, but the recurrence rate remains high. Superficial fibromatosis typically presents as subcutaneous nodules caused by rapid myofibroblast proliferation followed by slow involution to dense acellular fibrosis. In this study, we demonstrate that fibromatosis stem cells (FSCs) can be isolated from palmar nodules but not from cord or normal palm tissues. We found that FSCs express surface markers such as CD29, CD44, CD73, CD90, CD105, and CD166 but do not express CD34, CD45, or CD133. We also found that FSCs are capable of expanding up to 20 passages, that these cells include myofibroblasts, osteoblasts, adipocytes, chondrocytes, hepatocytes, and neural cells, and that these cells possess multipotentiality to develop into the three germ layer cells. When implanted beneath the dorsal skin of nude mice, FSCs recapitulated human fibromatosis nodules. Two weeks after implantation, the cells expressed immunodiagnostic markers for myofibroblasts such as α-smooth muscle actin and type III collagen. Two months after implantation, there were fewer myofibroblasts and type I collagen became evident. Treatment with the antifibrogenic compound Trichostatin A (TSA) inhibited the proliferation and differentiation of FSCs in vitro. Treatment with TSA before or after implantation blocked formation of fibromatosis nodules. These results suggest that FSCs are the cellular origin of fibromatosis and that these cells may provide a promising model for developing new therapeutic interventions.

## Introduction

Musculoskeletal fibromatosis is a condition that presents as benign soft tissue tumors with an aggressive course. This disease can be divided into superficial (fascial) and deep (musculoaponeurotic) groups, and both share a common histopathological appearance. The superficial group tumors are typically small, slow-growing lesions and include palmar fibromatosis (Dupuytren's disease) [1], plantar fibromatosis [2], penile fibromatosis (Peyronie's disease) [3], and infantile digital fibroma [4]. The typical clinical picture of superficial fibromatosis involves the formation of subcutaneous fibromatosis nodules that progress slowly to involve the skin and deep structures or to form a cord, which causes contractures. In contrast to the superficial group of fibromatosis, the lesions of deep fibromatosis are often large, more aggressive, and possible faster growing. Deep fibromatosis includes aggressive fibromatosis (desmoid tumor) [5], infantile myofibromatosis, fibromatosis colli, and aggressive infantile fibromatosis.

The main components of fibromatosis are myofibroblasts, which exhibit features of smooth muscle cells and fibroblasts surrounded by abundant collagen material. α-Smooth muscle actin (α-SMA) and type III collagen are specific markers of myofibroblasts in fibromatosis [6]; [7]. Despite recent advances in understanding the biochemical and cellular processes of fibromatosis, the precursor cells of myofibroblasts and uncontrolled growth behavior of fibromatosis remain elusive, and the pathogenesis of fibromatosis remains unclear [6]; [8]–[11]. The main treatment option for fibromatosis is surgical excision; fibromatosis does not metastasize [12], but local regional control remains challenging with excision alone because of a high local recurrence rate [2]; [13]–[15].

Adult stem cells, like pluripotent stem cells, have the ability to self-renew and to differentiate into multiple lineage cells including bone, fat, cartilage [16], and nonmesenchymal tissues such as neurons [17] and hepatocytes [18]. Stem cells are known to exist in various tissues, although it is unclear whether stem cells can be isolated from fibromatosis tissues. Palmar fibromatosis is the most common type of fibromatosis and shares the same clinical course with other fibromatosis [19], and we therefore conducted experiments to isolate stem cells from palmar fibromatosis and refer to these cells here as fibromatosis-derived stem cells (FSCs).

A hallmark of palmar fibromatosis is its clinical course, which is divided into three stages [20]. The earliest proliferative stage is characterized by nodule formation with hypercellular areas full of proliferating myofibroblasts and newly formed capillaries. In the involutional stage, the hypercellular areas have fewer cells and the expression of type I collagen increases. In the residual stage, nodules are replaced by dense acellular fibrosis [21]. To our knowledge, no animal model of fibromatosis nodules has been reported. We postulated that a murine model of fibromatosis nodule could be developed by implanting FSCs into the back of nude mice. The characteristic properties of FSCs in this murine model include their unique ability to recapitulate the natural course of human palmar fibromatosis.

To identify an early nonsurgical treatment for fibromatosis nodules before they progress to the stage where only surgical excision can be applied, our group investigated the potential antifibrogenic effect of trichostatin A (TSA), a histone deacetylase inhibitor (HDACi). It has been reported that treating various tumor cells with TSA can induce cell differentiation, cell apoptosis, or necrosis. TSA is thought to have antifibrogenic potential by inhibiting α-SMA [22] and has been tested as a promising therapeutic agent in hepatic fibrosis [23]. However, the therapeutic potential of TSA in treating fibromatosis has not been investigated. In this study, we used our murine model to examine the therapeutic effect of TSA on FSCs in vitro and in vivo.

## Materials and Methods

### Isolation and expansion method

This research followed the tenets of the Declaration of Helsinki. Fibromatosis tissues were obtained from six patients receiving excision for palmar fibromatosis after they provided written informed consent (Table 1). The study protocol and written informed consent forms were approved by the Institutional Ethics Committee/Institutional Review Board of Taipei Veterans General Hospital, Taiwan.

**Table 1:** Details of the donors with palmar fibromatosis-derived stem cells.

Donor no.	Age (years)/ gender	Duration(months)	Joint of Contracture	Source of stem cells
1	69/female	7	MCP, PIP	Nodule
2	83/male	5	MCP	Nodule
3	49/male	4	MCP	Nodule, Cord
4	85/male	7	MCP, PIP	Nodule, Cord
5	46/male	10	MCP, PIP	Nodule,
6	83/male	5	MCP	Nodule

MCP joint: Metacarpalphalangeal joint.

PIP joint: Proximal interphalangeal joint.

The tissues were washed repeatedly in phosphate-buffered saline (PBS; Gibco BRL, Grand Island, NY); the fat tissues were scraped off carefully, leaving only the nodule and cord portion of the palmar fibromatosis tissues to be used in the experiment. The tissues were collected by centrifugation and digested with 3 mg/ml collagenase for 3 h. The nucleated cells were then plated at clonal density and cultured in α-minimal essential medium (α-MEM, Invitrogen, Carlsbad, CA) containing 10% fetal bovine serum (FBS; Invitrogen; lot selected for rapid growth), 100 U/ml penicillin (Invitrogen), 100 µg/ml streptomycin (Invitrogen), and 250 ng/ml amphotericin B (Invitrogen). The cells were fed every 2 days by changing to fresh growth medium and propagated every 4 days at a 1∶5 split before cell growth reached 80% confluence.

To track the cellular fate of FSCs in our murine model of fibromatosis nodules, FSCs were transduced with a lentiviral green fluorescent protein (GFP) vector followed by antibiotic selection. GFP-expressing FSCs delivered in growth factor-reduced Matrigel (BD Biosciences, Bedford, MA) were injected subcutaneously into nude mice, and the implants were harvested at the times indicated, fixed in paraformaldehyde, and processed for paraffin sections.

### Flow cytometric analysis

To analyze the cell surface expression of typical marker proteins, FSCs were harvested in 5 mmol/l EDTA in PBS. Cells were incubated with the following anti-human antibodies: CD34–phycoerythrin (PE), CD73–PE (also referred to as SH3 and SH4), CD90–fluorescein isothiocyanate (FITC, Becton Dickinson, San Jose, CA), CD29–FITC, CD44–FITC, CD45–FITC (Beckman Coulter, Krefeld, Germany), CD133–PE (Miltenyi Biotec, Bergisch Gladbach, Germany), CD105–FITC(SH2), or CD166–FITC (ImmunoKontact, AMS Biotechnology, Wiesbaden, Germany). Mouse isotype antibodies (Becton Dickinson and Beckman Coulter) were used as controls. Ten thousand labeled cells were acquired and analyzed using a FACScan flow cytometry running CellQuest software (Becton Dickinson).

### Cell proliferation assay

The cytotoxicity effect of TSA on FSCs was measured with a cell proliferation test kit using 3-(4,5-dimethylthiazol-2-yl)-2,5- diphenyltetrazolium bromide (MTT, Sigma, St Louis, MO) to determine the inhibitory concentrations (IC_50_-values) of TSA. The cells were seeded at 2000 cells/well in a 96-well culture plate and incubated with different concentrations of TSA (0, 10, 100, 200, 400, 600, 800, and 1000 nM). The absorbance after incubation with MTT for 4 h at 37°C in 5% CO_2_ was measured with an enzyme-linked immunosorbent assay (ELISA) plate reader after 7 days. Cell numbers were determined using the optical density (OD) value at a test wavelength of 560 nm. The IC_50_-values were determined and used as an indicator of proliferation inhibition.

### Differentiation protocols

The FSCs from all donors were induced under the following culture conditions. The non-stem cell lines 293T cells were used as negative control cells [24].

- Osteogenic differentiation medium comprised α-MEM supplemented with 10% FBS, 50 µg/ml ascorbate-2 phosphate (Nacalai, Kyoto, Japan), 10^−8^ M dexamethasone (Sigma), and 10 mM β-glycerol phosphate (Sigma) [25].
- Adipogenic differentiation medium comprised α-MEM supplemented with 10% FBS, 50 µg/ml ascorbate-2 phosphate, 10^−7^ M dexamethasone, 50 µg/ml indomethacin (Sigma), 0.45 mM 3-isobutyl-1-methylxanthine (Sigma), and 10 µg/ml insulin (Sigma) [25].
- Cells were induced in a defined chondrogenic induction medium comprising serum-free DMEM high-glucose (DMEM-HG) supplemented with ITS^+^ Premix (BD Biosciences, Bedford, MA: 6.25 µg/ml insulin, 6.25 µg/ml transferrin, 6.25 µg/ml selenious acid, 1.25 mg/ml bovine serum albumin (BSA), 5.35 mg/ml linoleic acid), 10^−7^ M dexamethasone (Sigma), 50 µg/ml ascorbate-2-phosphate (Sigma), and 10 ng/ml transforming growth factor (TGF)-β1. The pelleted cells were incubated at 37°C in 5% CO_2_. Within 12–24 h of incubation, the cells formed an essentially spherical cluster that did not adhere to the walls of the tube. The medium was changed every 3 days. Cells were then used for histochemical staining and immunofluorescence study after the morphological features of differentiation appeared. Cells were washed with PBS, fixed in 3.7% paraformaldehyde for 10 min at room temperature, and stained with Alizarin red-S following osteogenic differentiation to reveal osteogenic differentiation. Cells treated under adipogenic and chondrogenic culture conditions were stained with Oil red-O and Alcian blue to show adipogenic and chondrogenic differentiation, respectively. Type II collagen staining was identified by immunohistochemistry [25].
- To induce hepatogenic differentiation, cells at 5×10^5^ cells/100-mm dish were serum deprived for 2 days, cultured in α-MEM supplemented with 20 ng/ml epidermal growth factor (EGF, Sigma) and 10 ng/ml basic fibroblast growth factor (bFGF,Sigma), and then subjected to induction by a 2-step protocol. Differentiation was induced by treating cells with step-1 differentiation medium comprising α-MEM supplemented with 20 ng/ml hepatocyte growth factor (Sigma), 10 ng/ml bFGF, and 0.61 g/l nicotinamide (Sigma) for 7 days, followed by treatment with step-2 maturation medium, comprising α-MEM supplemented with 20 ng/ml oncostatin M (R&D Systems), 1 mol/l dexamethasone, and 50 mg/ml ITS^+^ Premix (BD Biosciences: 6.25 µg/ml insulin, 6.25 µg/ml transferrin, 6.25 µg/ml selenious acid, 1.25 mg/ml BSA, 5.35 mg/ml linoleic acid) for 7 days [18].
- To induce neural differentiation, cells at 3×10^5^ cells/100-mm dish were pretreated with α-MEM supplemented with 10% FBS, 10% fetal calf serum, 10^−3^ M mercaptoethanol (Sigma), and 10^−7^ M all-*trans*-retinoic acid (Sigma) for 24 h, and then deprived of serum for 5 days [17].

### Reverse transcription (RT) and real-time polymerase chain reaction (PCR) analysis

Total RNA was prepared using TRIzol reagent (Invitrogen, Australia). For first-strand cDNA synthesis, random sequence primers were used to prime RT reactions and synthesis was performed using SuperScript^TM^ III Reverse Transcriptase (Invitrogen, Carlsbad, CA). cDNA was synthesized from total RNA using M-MuLV reverse transcriptase. PCR was performed with cDNA as the template in a 30-μl reaction mixture containing specific primer pairs (Table 2). Negative controls were used for each primer by replacing cDNA by distilled water on the one hand and replacing cDNA by RNA in order to exclude contamination with genomic DNA (Fig. S2). PCR was performed using Taq DNA Polymerase Recombinant (Invitrogen), and each cycle comprised the following steps: denaturation for 45 s at 94°C, annealing for 1 min at 51–58°C, and 90 s of elongation at 72°C. Glyceraldehyde-3-phosphate dehydrogenase (GAPDH) was used to normalize input template cDNA to analyze relative gene expression. The reaction products were resolved by electrophoresis on a 1.5% agarose gel and visualized with ethidium bromide.

**Table 2:** Primers used for reverse transcription-polymerase chain reaction (RT-PCR) analysis.

Gene	Sense primer	Anti-sense primer
LPL	GGTCGAAGCATTGGAATCCAG	TAGGGCATCTGAGAACGAGTC
PPARγ2	CCTATTGACCCAGAAAGCGATTC	GCATTATGAGACATCCCCACTGC
OP	CTAGGCATCACCTGTGCCATACC	CAGTGACCAGTTCATCAGATTCATC
RUNX2	GTTTGTTCTCTGACCGCCTC	CCAGTTCTGAAGCACCTGA
COL2A1	CCAGGACCAAAGGGACAGAAAG	TTCACCAGGTTCACCAGGATTG
βIII-TUB	CGAGACCTACTGCATCGACA	GGGATCCACTCCACGAAGTA
NES	TTCCCTTCCCCCTTGCCTAATACC	TGGGCTGAGCTGTTTTCTACTTTT
AFP	TGCAGCCAAAGTGAAGAGGGAAGA	CATAGCGAGCAGCCCAAAGAAGAA
ALB	TGCTTGAATGTGCTGATGACAGGG	AAGGCAAGTCAGCAGGCATCTCATC
GAPDH	ATATTGTTGCCATCAATGACC	GATGGCATGGACTGTGGTCATG

LPL, lipoprotein lipase; PPARγ, peroxisome proliferator-activated receptor-γ; OP, osteopontin; RUNX2, runt-related transcription factor 2; COL2A1, α-1 type II collagen; βIII-TUB, β-tubulin III; NES, nestin; AFP, α-fetoprotein; ALB, albumin; GAPDH, Glyceraldehyde-3-phosphate dehydrogenase.

Real-time amplification of the genes was performed using the ABI Assays on Demand primers^©^ and SYBR green^®^ universal PCR Master Mix on the ABI 7500 real-time PCR instrument according to the manufacturer's instructions (Applied Biosystems, Foster City, CA). To check the efficiency of PCR amplification and cDNA synthesis, GAPDH was used as an internal control (Table 3). Analysis of the results was performed using the software supplied with the machine using the Δ_CT_ method.

**Table 3:** Primers used for real-time reverse transcription-polymerase chain reaction analysis.

Gene	Primer length	T_m_	Sense primer	Anti-sense primer
Col1A2	20	51.78	GACATGCTCAGCTTTGTGGA	CTTTCTCCACGTGGTCCTCT
Col3A1	20	50.2	GGAGAATGTTGTGCAGTTTG	AGGACCAGTAGGGCATGA
α-SMA	20	53.7	CATCATGCGTCTGGATCTGG	GGACAATCTCACGCTCAGCA
GAPDH	21	52.57	ATATTGTTGCCATCAATGACC	GATGGCATGGACTGTGGTCATG

Col1A2, α2 type I collagen; Col3A1, α1 type III collagen; α-SMA, α-smooth muscle actin; GAPDH, Glyceraldehyde-3-phosphate dehydrogenase.

### In vivo murine model of fibromatosis nodule

The animal care and experimental protocols were in accordance with the institutional animal welfare guidelines of Taipei Veterans General Hospital. All procedures involving animals were approved by the institutional animal care and use committee of Taipei Veterans General Hospital (98-081). Matrigel implants were used to injected the mice with benign tumor stem cells to recapitulate benign tumors [26], and we used this method to recapitulate the development of fibromatosis nodules. The containment of cells within Matrigel in the implant helps entrap the cells within the injection site. The murine model of fibromatosis nodules was created by implantation of FSCs in 4–6-week-old male athymic nu/nu mice. In brief, 1.0×10^6^ FSCs suspended in 200 µl Matrigel were injected subcutaneously into the back of the mouse, and each mouse received 4 injections. Animals were sacrificed and the Matrigel implants were harvested at 7, 14, 28, and 56 days. The Matrigel implants were fixed in 3.7% paraformaldehyde for immunohistochemistry and immunofluorescence staining.

In previous studies, TSA was given subcutaneously at a dose of 0, 1, 2, or 5 mg/kg every day in murine models of retroperitoneal neuroblastoma [27], arthritis [28], and endometriosis [29]. In the current study, TSA (2 mg/kg body weight) dissolved in 40 µl of dimethyl sulfoxide (DMSO) was injected subcutaneously daily for 1 week. To maintain the integrity of the Matrigel implants, TSA was injected subcutaneously between the Matrigel implants.

### Immunohistochemistry and immunofluorescence staining

Paraffin-embedded sections were deparaffinized in xylene, dehydrated through graded alcohols from 100% to 70%, and then rinsed in ddH_2_O to remove the organic solution. Antigen retrieval was achieved by boiling the slides in 10 mM sodium citrate buffer pH 6.0 at 99°C for 30 min. The slides were then cooled to room temperature, rinsed in PBS, and blocked with 3% H_2_O_2_; nonspecific staining was blocked with PBS containing 5% heat-inactivated FBS for 30 min at room temperature. A mouse monoclonal antibody against human type II collagen (Daiichi Fine Chemical Co., Toyama, Japan) was used as the primary antibody. Polymer–horseradish peroxidase (HRP, BioGenex, San Ramon, CA) was used as the secondary antibody, and diaminobenzidine (DAB; BioGenex) was applied until the color appeared. The sections were counterstained with Harris hematoxylin (Sigma-Aldrich, St. Louis, MO) and mounted. Sections of hematoxylin and eosin (H&E)-stained Matrigel implants were observed on an Olympus AX80 light microscope and the images were quantified using ImageJ software.

For immunofluorescence staining, primary antibodies against α-SMA (Sigma-Aldrich), type III collagen (Abcam, Cambridge, UK), type I collagen (Abcam), glial fibrillary acidic protein (GFAP; Cell Signaling Technology, Beverly, MA), β-tubulin III (Santa Cruz Biotechnology, Santa Cruz, CA), GFP (Abcam), and albumin (Santa Cruz Biotechnology) were placed on slides at appropriate dilutions. The sections were then incubated with DyLight 594-conjugated goat anti-rabbit IgG, DyLight 488-conjugated goat anti-rabbit IgG, DyLight 594-conjugated goat anti-mouse IgG, or DyLight 488-conjugated donkey anti-goat IgG secondary antibodies (all from Jackson ImmunoResearch Laboratories Inc., West Grove, PA). The sections were counterstained with 4,6-diamidino-2-phenylindole (DAPI; Vector Labs, Burlingame, CA) for nuclear (blue) fluorescent staining.

### Statistical analysis

All statistical analyses were performed using SAS statistical software (version 6.12, SAS Institute, Cary, NC) or SPSS software (version 8.0, SPSS, Chicago, IL). The results are presented as mean ± SD. The significance of differences between the experimental and control groups was determined by analysis of variance, and a p value<0.05 by Student's *t* test was considered significant.

## Results

### Isolation of FSCs by enzymatic digestion and plastic adherence

**Figure 1:**
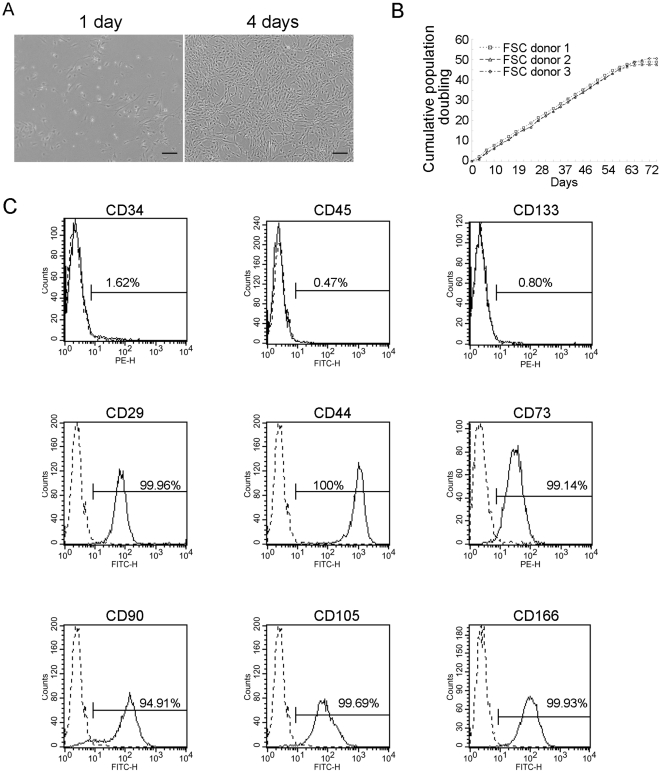
Characterization of FSCs. (A) Morphology of FSCs 1 and 4 days after seeding. Bars = 200 µm. (B) Proliferation curve of FSCs from different donors. Cumulative population doubling curves vs. time of in vitro culture are based on 3- or 4-day split passages of FSCs at a 1∶5 ratio. (C) Flow cytometric analysis of the surface protein profile of FSCs at passage 4; the dashed line represents the isotype IgG and the solid line indicates the antibody. All experiments were repeated with FSCs from three different donors.

Isolation of FSCs was based on the measures of enzyme digestion and plastic adherence [25]. The cell mixture was obtained by adding collagenases to tissues of palmar fibromatosis. Enrichment of FSCs was achieved by removing nonadherent cells when changing to fresh growth medium at 2-day intervals. The FSCs were plastic adherent with spindle-shaped morphology (Fig. 1A). The cells were propagated every 4 days at a 1∶5 split when they reached 80% confluence. The doubling times of FSCs were estimated at 40–48 h at a seeding density of 4000 cells/cm^2^. The FSCs could be expanded in culture for up to 20 passages while maintaining their proliferative and undifferentiated status in the earliest passages. The rate of replication of the FSC cultures plateaued after about 10 weeks after 40 population doublings (PD), when subcultivation became impossible (Fig. 1B). The isolation efficiency of FSCs was much higher in nodules compared with cords or normal tissues obtained from the same palm (Table 1), suggesting that the nodules contained more FSCs than the cords.

### Flow cytometric analysis of FSCs

For phenotypic characterization of the FSCs, we first used flow cytometry to examine the expression of different CD surface antigens at passages 3 to 5. These cells from six individual donors were consistently positive for several putative mesenchymal stem cell (MSC) markers, such as CD29, CD44, CD73, CD90, CD105, and CD166, but were negative for markers of early or differentiated hematopoietic cells such as CD34, CD45, and CD133 (Fig. 1C). These results suggested that FSCs expressed the same surface markers as MSCs from bone marrow and other tissues.

### Myofibroblast differentiation of FSCs

**Figure 2:**
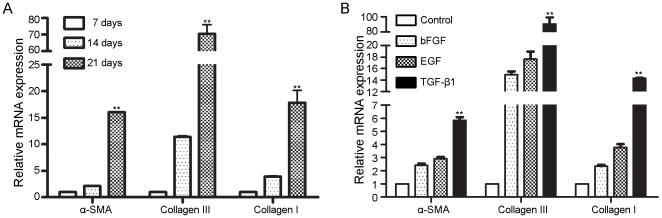
Confluence-induced and growth factor-induced fibrogenic potential of FSCs. (A) FSCs were seeded at 4000 cells/cm^2^, cultured, and harvested after 7, 14, and 21 days, and the relative mRNA levels of α-SMA, collagen type III, and collagen type I were evaluated by quantitative RT-PCR. (B) FSCs were treated with or without (Control) 10 ng/ml bFGF, 10 ng/ml EGF, or 10 ng/ml TGF-β1 for 7 days. Cells were harvested, and the relative mRNA levels of α-SMA, collagen type III and collagen type I was evaluated by quantitative RT-PCR. All transcript levels were normalized to GAPDH transcript production. Statistical significance is presented as **, p<0.01 compared with the other groups. All experiments were repeated with FSCs from three different donors.

We first investigated whether FSCs differentiate into myofibroblasts in vitro by analyzing the expression of α-SMA and type III collagen. Quantitative RT-PCR demonstrated that culturing FSCs increased the expression of genes such as α-SMA, Col3A1, and Col1A3 as the time of culture continued (Fig. 2A), suggesting spontaneous differentiation of FSCs into myofibroblasts. We then studied whether fibrosis-related growth factors, such as bFGF, EGF, and TGF-β1 [30]–[32], induced myofibroblast differentiation. All of the growth factors examined here induced myofibroblast differentiation, and TGF-β1 had the strongest fibrogenic effect (Fig. 2B). These results indicated the myofibroblast differentiation potential of FSCs and the important role of FSCs in the development of fibromatosis.

### Differentiation potential of FSCs

**Figure 3:**
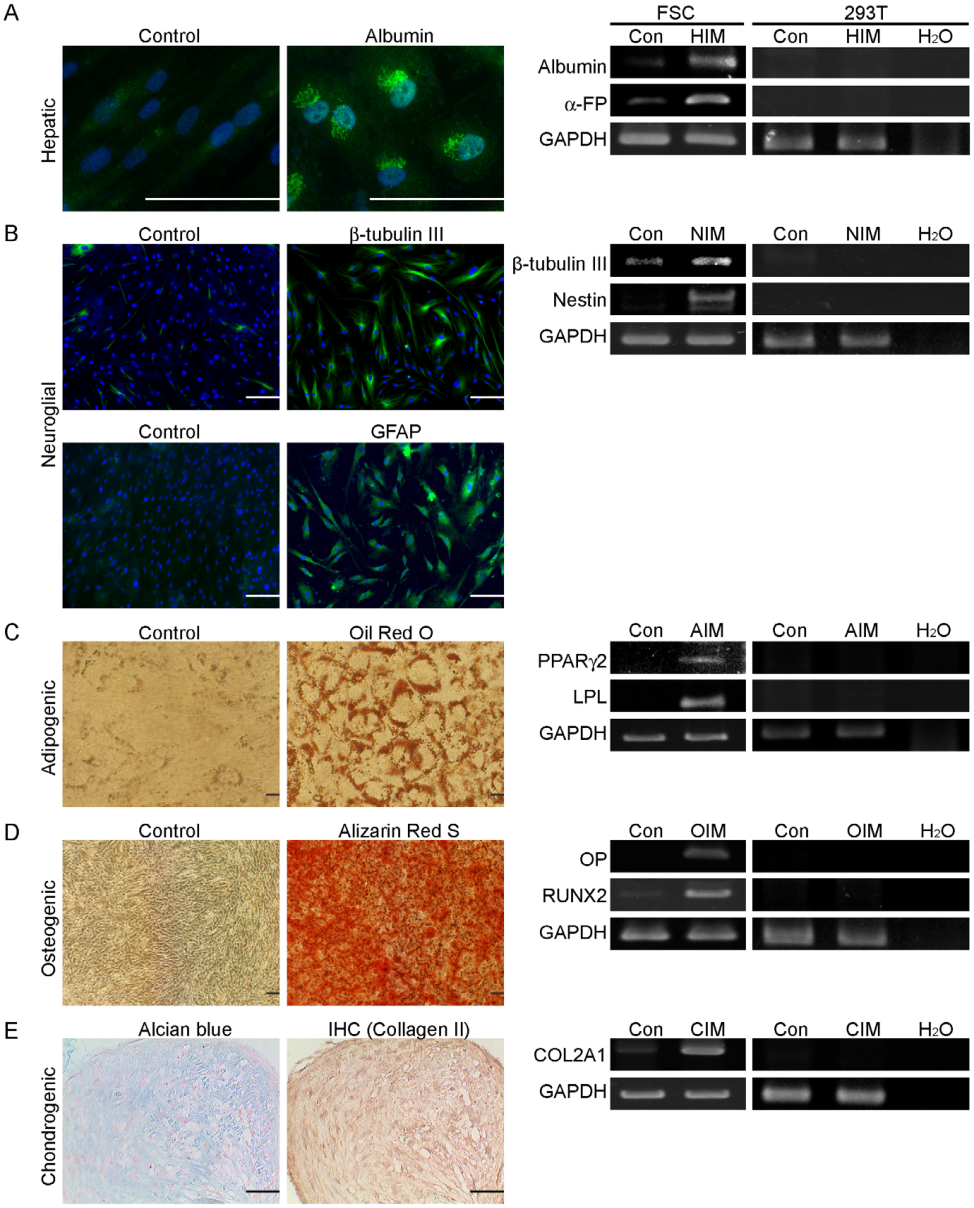
Differentiation potential of FSCs. (A) Hepatic potential of FSCs. Microscopic images showing morphology and albumin staining of hepatic differentiation at 14 days (HIM). Expression of hepatic genes after 14 days of hepatic differentiation (HIM) in FSCs as demonstrated by RT-PCR. (B) Neuroglial potential of FSCs. Microscopic images showing β-tubulin III and GFAP staining for neuroglial differentiation at 14 days (NIM). Expression of neuroglial genes after 14 days of neuroglial differentiation (NIM) in FSCs as demonstrated by RT-PCR. (C) Adipogenic potential of FSCs. Microscopic images showing Oil red-O staining of control cells (Con) and adipogenic differentiation at 21 days (AIM). Expression of adipogenic genes after 7 days of adipogenic differentiation (AIM) in FSCs as demonstrated by RT-PCR. (D) Osteogenic potential of FSCs. Microscopic images showing Alizarin red-S staining of control cells (Con) and osteogenic differentiation at 21 days (OIM). Expression of osteogenic genes after 7 days of osteogenic differentiation (OIM) in FSCs as demonstrated by RT-PCR. (E) Chondrogenic potential of FSCs. Alcian blue staining and immunohistochemical staining of type II collagen at 21 days of chondrogenic differentiation. Expression of chondrogenic genes after 7 days of chondrogenic differentiation (CIM) in FSCs as demonstrated by RT-PCR. All experiments were repeated with FSCs from three different donors. All experiments were performed with FSCs at passages 5–8. The non-stem cell lines 293T cells were used as negative control cells. Bars = 100 µm. (Con: no induction control).

To study the differentiation potential of FSCs further, FSCs were induced to differentiate along the hepatic, neuroglial, adipogenic, osteogenic, and chondrogenic lineages. Hepatic differentiation was demonstrated clearly by albumin staining at 2 weeks of differentiation (Fig. 3A). Significantly greater expression of albumin and α-fetoprotein genes was detected at 2 weeks of differentiation compared with the control culture condition (Fig. 3A). Neuroglial differentiation was demonstrated clearly by the accumulation of β-tubulin III and GFAP at 2 weeks of differentiation (Fig. 3B). Significantly greater expression of β-tubulin III and nestin genes was detected at 2 weeks of differentiation compared with the control culture condition (Fig. 3B). Adipogenic differentiation was demonstrated clearly by the accumulation of Oil red-O-stained lipid vesicles at 3 weeks of differentiation, whereas cells cultured in control growth medium were negative for Oil red-O staining (Fig. 3C). Significantly greater expression of peroxisome proliferator-activated receptor-γ (PPARγ) and lipoprotein lipase (LPL) genes was detected at 3 weeks of differentiation compared with the control culture condition (Fig. 3C). Osteogenic differentiation was demonstrated in cells stained positive for Alizarin red-S at 2 and 3 weeks of osteogenic differentiation, whereas cells grown in control growth medium were not stained by Alizarin red-S (Fig. 3D). RT-PCR demonstrated further the expression of osteopontin (OP) and runt-related transcription factor 2 (RUNX2) at 14 days of osteogenic differentiation, whereas cells grown in control medium did not express OP (Fig. 3D). At 3 weeks of chondrogenic differentiation, Alcian blue staining and type II collagen immunohistochemistry revealed depositions of glycosaminoglycan and type II collagen in pelleted cultured cells, respectively (Fig. 3E). RT-PCR analysis also revealed the expression of cartilage oligomeric protein (COMP) and COL2A1 genes (Fig. 3E). All of these results demonstrated the differentiation potential of FSCs.

### Formation of fibromatosis-like nodules by FSCs in immunodeficient mice

**Figure 4:**
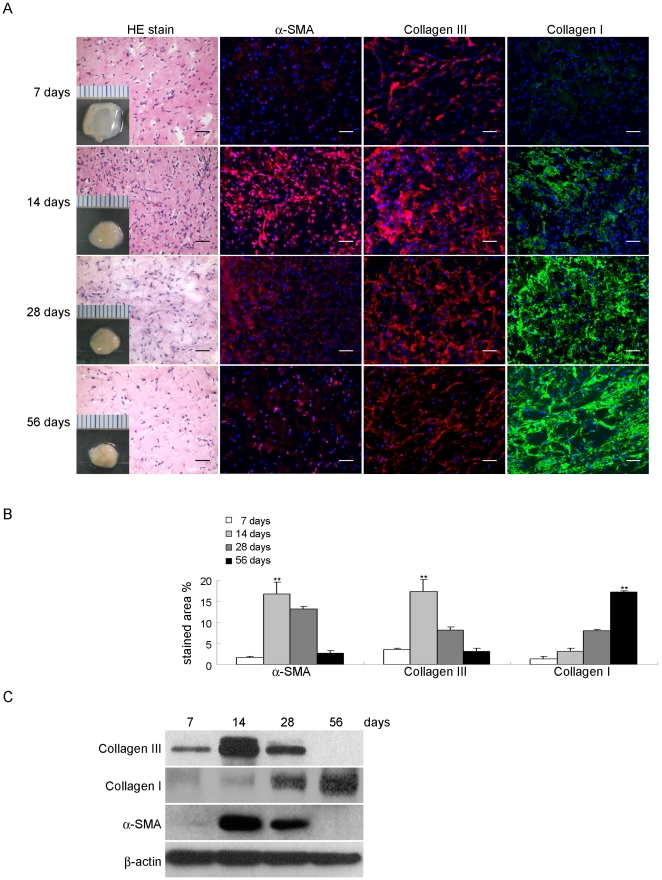
Formation of fibromatosis nodules by FSCs in vivo and expression of multiple fibrotic proteins at different stages. (A) FSCs were delivered in Matrigel and implanted beneath the dorsal skin of nude mice. Nodule formation was seen at 14 days and involution at 56 days. Small cubes: macroscopic views of the implants after 7, 14, 28, and 56 days in vivo. Scale = 1 mm. H&E staining and immunofluorescence analysis of α-SMA, types III and I collagen. Bars = 50 µm. (B) The percentages of stained areas. (C) Western blotting for α-SMA, types III and I collagen. Data are shown as mean ± SD (n = 3). Statistical significance is presented as **, p<0.01 compared with other groups. All experiments were repeated with FSCs from three different donors.

To determine whether FSCs can form fibromatosis nodules in our murine model, the cells were delivered in Matrigel and implanted subcutaneously beneath the dorsal skin of nude mice for 56 days. Staining of the sections showed an increase in the accumulation of α-SMA and type III collagen, myofibroblast density, and vessel formation at 7 and 14 days (Fig. 4A, 4B). This was similar to the histological picture of the composition of nodules in the proliferative phase of human fibromatosis (Fig. S1). By contrast, Matrigel alone or bone marrow MSCs implanted in Matrigel did not form fibromatosis nodules and seemed to be decomposing (data not shown). The next objective was to determine whether FSCs recapitulated the involutional and residual phases of fibromatosis nodules. Staining of the sections with H&E and immunofluorescence from 28 to 56 days after implantation of Matrigel showed decreased myofibroblast density and increased accumulation of type I collagen (Fig. 4A, 4B). This was consistent with the reduced number of cells seen during the involution of human fibromatosis nodules (Fig. S1). A gross view showed that the Matrigel was becoming firm and opaque over time because the Matrigel was replaced by type I collagen (Fig. 4A). The results of western blotting for α-SMA and types III and I collagen were also consistent with the immunofluorescence findings (Fig. 4C).

### Differentiation of GFP-labeled FSCs into myofibroblasts in vivo

**Figure 5:**
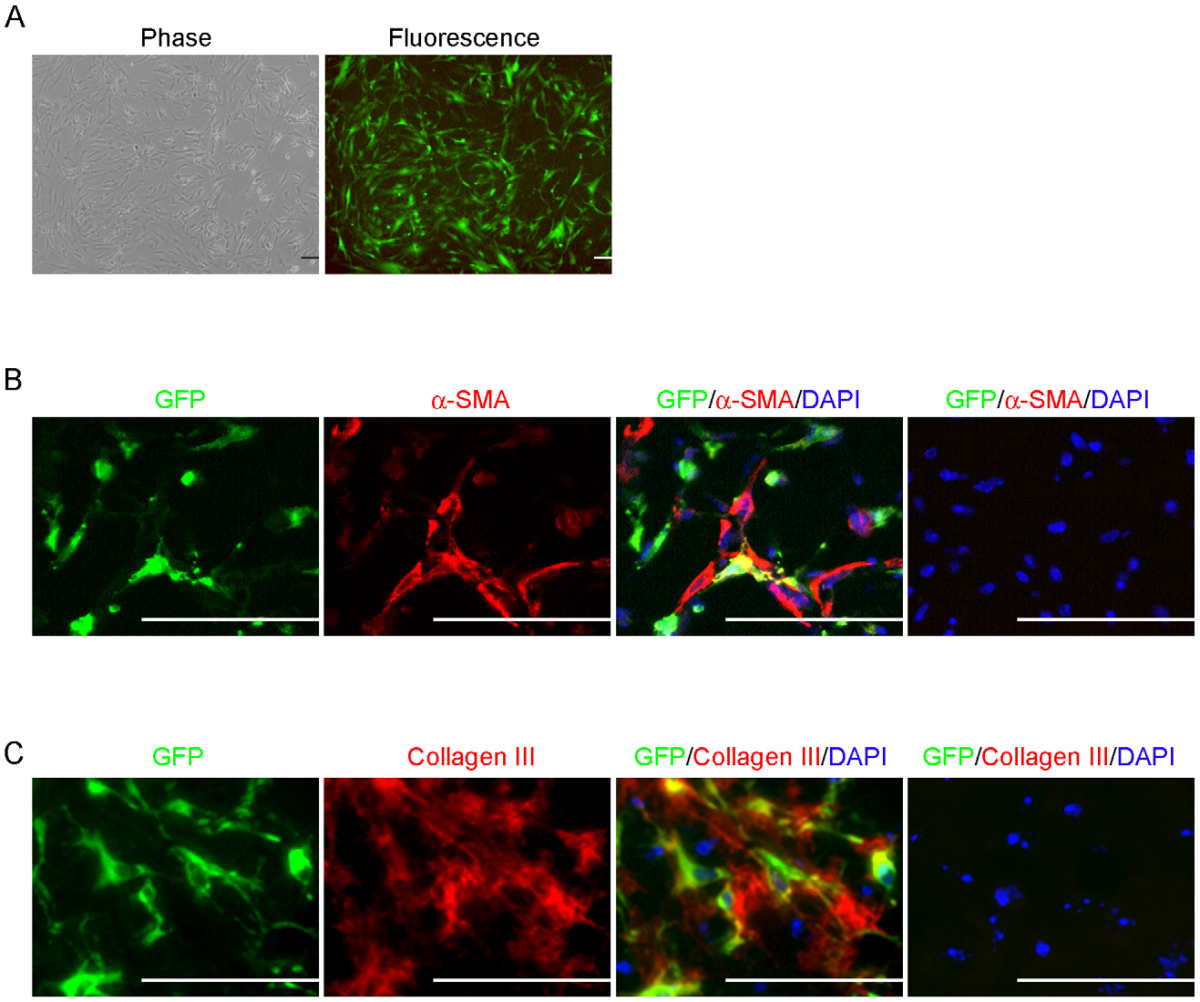
Formation of fibromatosis nodules by GFP-labeled FSCs in vivo. (A) FSCs were infected with a lentiviral vector carrying the GFP gene. Images show the morphology and GFP fluorescence after selection for 7 days. (B–C) GFP-labeled FSCs were delivered in Matrigel and implanted beneath the dorsal skin of nude mice. Microscopic views of the implants after 14 days. (B) Immunofluorescence for GFP (green), α-SMA (red), and DAPI (blue). (Right panel) Absence of staining with the primary antibody for GFP and α-SMA was used as a negative control. (**C**) Sections were also stained for GFP (green), type III collagen (red) and DAPI (blue). (Right panel) Absence of staining with the primary antibody for GFP and type III collagen was used as a negative control. Bars = 50 µm. All experiments were repeated with FSCs from three different donors.

To examine the cellular fate of FSCs in our murine model of fibromatosis nodules, FSCs were transduced with a lentiviral vector carrying the GFP gene (Fig. 5A). Costaining for the GFP-tracking marker and myofibroblast markers 14 days after implantation with Matrigel showed that GFP-labeled cells were also positive for α-SMA and type III collagen (Fig. 5B, 5C). These data indicated that the newly formed fibromatosis nodules were derived from human FSCs.

### Inhibition by TSA of FSC proliferation and differentiation in vitro

**Figure 6:**
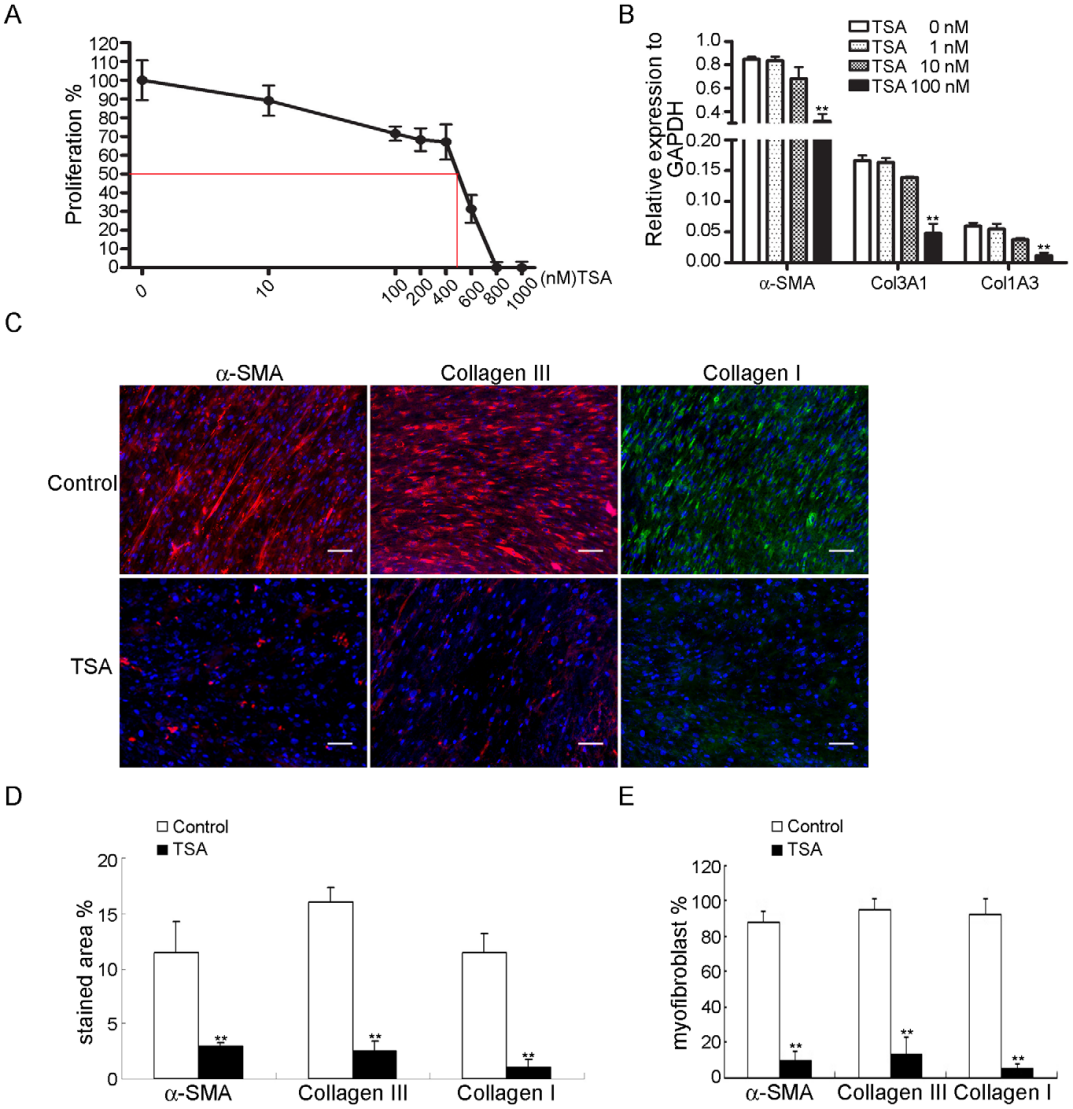
Inhibition by TSA of proliferation and differentiation of FSCs in vitro. (A) FSCs seeded at 2000 cells/well in 96-well plates were treated with TSA at the indicated concentrations for 7 days. The IC_50_-value was measured. (B) FSCs were cultured in the presence of the indicated concentration of TSA, and quantitative RT-PCR analysis was performed at 14 days. (C) Immunofluorescence for α-SMA and types III and I collagen. FSCs were cultured with or without TSA, and immunofluorescence analysis was performed at 14 days. Bars = 50 µm. (D) The percentages of stained areas. (E) The percentages of myofibroblasts. Data are shown as mean ± SD (n = 3). Statistical significance is presented as **, p<0.01 compared with other groups. All experiments were repeated with FSCs from three different donors.

Because TSA is a fibrogenic inhibitor, we next assessed the effects of TSA on the proliferation and fibrogenesis of FSCs. The IC_50_-value was 400–600 nM (Fig. 6A). Quantitative RT-PCR showed that treatment for 14 days with TSA at concentrations that did not induce cytotoxicity inhibited the expression of α-SMA, Col3A1, and Col1A3 mRNAs in a dose-dependent manner (Fig. 6B). Immunofluorescence also demonstrated that TSA inhibited the expression of α-SMA and type III collagen (Fig. 6C, 6D). These data suggested that TSA suppressed the proliferation and myofibroblast differentiation of FSCs in vitro (Fig. 6E).

### Inhibition by TSA of the ability of FSCs to form fibromatosis-like nodules in immunodeficient mice

**Figure 7:**
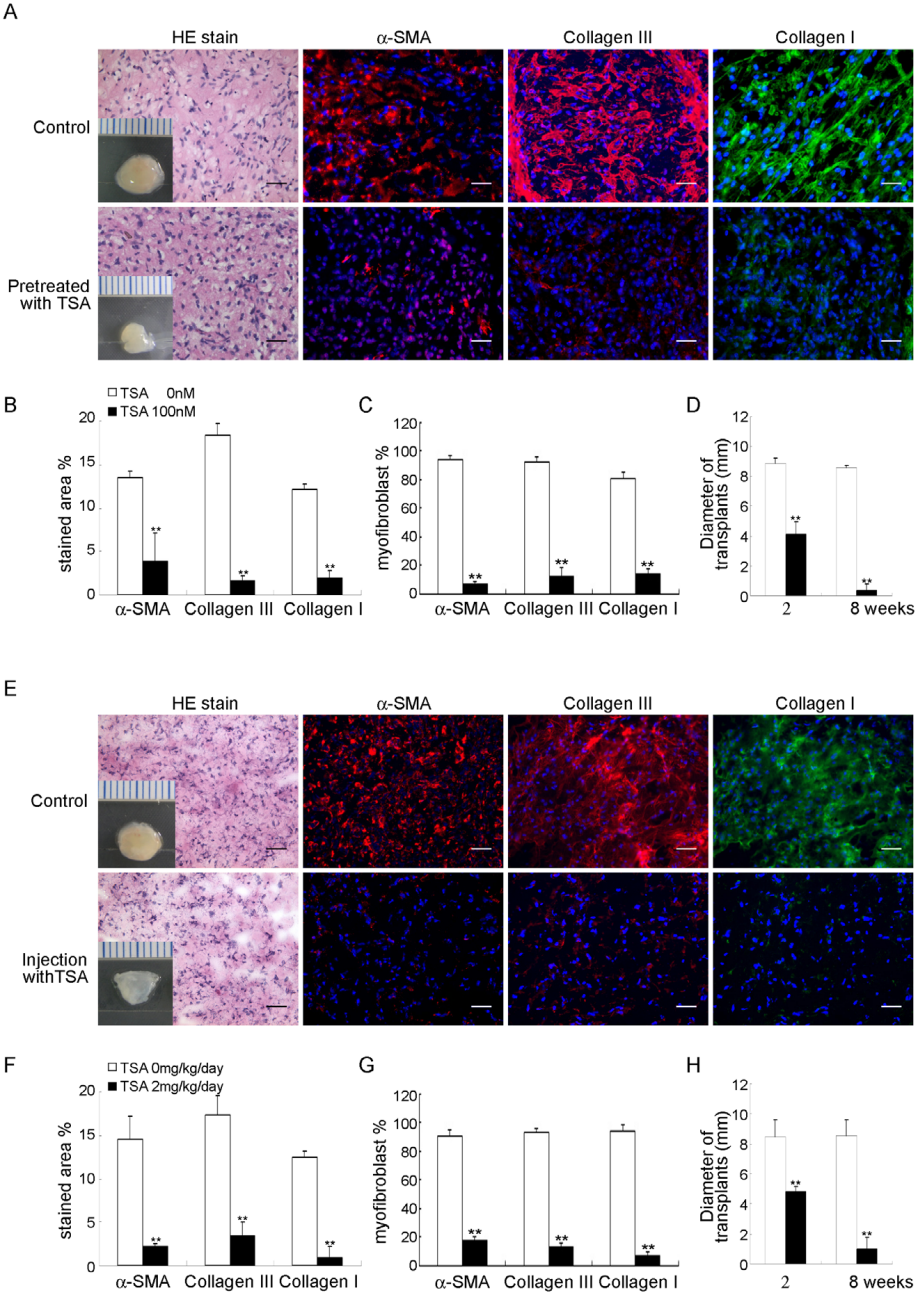
Inhibition by TSA of fibromatosis nodule formation by FSCs in this murine model. (A–D) FSCs were treated with 100 nM TSA for 3 days and then delivered in Matrigel implanted beneath the dorsal skin of nude mice. (A) Macroscopic views of the implants after 14 days in vivo (scale bar, 1 mm). H&E staining and immunofluorescence observations of α-SMA and types III and type I collagen were performed. Bars = 50 µm. (B) The percentages of stained areas. (C) The percentages of myofibroblasts. (D) The size of implants. Data are shown as mean ± SD (n = 3). **, p<0.01 denotes statistical significance. (E–H) FSCs were delivered in Matrigel and implanted beneath the dorsal skin of nude mice. After 7 days of implantation, TSA (2 mg/kg body weight) dissolved in 40 µl of dimethyl sulfoxide (DMSO) was injected subcutaneously daily for 1 week; the control group received daily subcutaneous injections of 40 µl of DMSO alone for 1 week. (E) Macroscopic views of the implants at 14 days of implantation in vivo (scale bar, 1 mm). H&E staining and immunofluorescence observations of α-SMA and types III and type I collagen. Bars = 50 µm. (F) The percentages of stained areas. (G) The percentages of myofibroblasts. (H) The size of implants. Data are shown as mean ± SD (n = 3). **, p<0.01 denotes statistical significance. All experiments were repeated with FSCs from three different donors.

To determine the effect of TSA on the ability of FSCs to form fibromatosis nodules in this murine model, FSCs were treated with TSA before or after implantation with Matrigel. TSA decreased the expression of α-SMA and the accumulation of type III and type I collagen compared with the control condition (Fig. 7A–7H). The Matrigel was not as firm, rounded and was going to be decomposed compared with the control condition (Fig. 7A–7H). These data suggested that TSA blocked the formation of fibromatosis nodules by FSCs in vivo.

## Discussion

The etiology of fibromatosis remains unclear and this disease has a high recurrence rate after surgery [14]. In our current study, we successfully isolated FSCs from tissues obtained from fibromatosis of the palm. The FSCs adopted a fibroblastic-like morphology and shared the surface protein profile of MSCs, and these cells could be subcultured up to 20 passages without significant loss of replication capacity. More importantly, FSCs expressed myofibroblast markers after being maintained in growth culture for a long time. FSCs are able to self-renew and differentiate along lineages including ectodermal, mesodermal, and endodermal lineages and therefore can be recognized as multipotent-like stem cells. The GFP-tracking system showed that the implanted FSCs formed fibromatosis nodules and differentiated into myofibroblasts in vivo. These results demonstrate that FSCs are the cellular precursors of fibromatosis. Although most isolated adult stem cells are excellent sources for tissue regeneration, these FSCs should be considered therapeutic targets or a promising animal model for the development of new therapeutic interventions.

At present, there is no animal model that represents the phenotypes and clinical course of human fibromatosis. We successfully developed a murine model of fibromatosis nodules by implanting human FSCs into immunodeficient mice. This model reflected the morphological phenotypes of fibromatosis nodules and recapitulated the clinical course of fibromatosis, including the proliferative, involutional, and residual stages. Because the GFP-tracking system showed that the myofibroblasts that synthesized α-SMA and type III collagen in nodules were derived from FSCs, which did not originally express α-SMA and type III collagen, our data suggest that this benign tumor can be recapitulated by these stem cells. Similar evidence has been reported in infantile hemangioma, which has been shown to develop after implantation of hemangioma stem cells in a murine model [26]. What makes our in vivo model unique is that only a small number of FSCs suspended in Matrigel were needed to produce the fibromatosis lesions in the absence of exogenous growth factors or supplements. This implies that there may be a genetic or epigenetic control in the FSCs that directs the cells to recapitulate fibromatosis.

Cell aggregation can induce differentiation into a variety of lineages such as osteogenesis, chondrogenesis, hepatic differentiation, neural differentiation, and insulin-producing cell differentiation [33]; [34], and this may help explain why FSCs spontaneously differentiated into myofibroblasts. FSCs cultured in growth medium formed aggregates, which may help to induce myofibroblast differentiation. However, the detailed mechanisms underlying this aggregation-induced differentiation remain elusive.

The existence of FSCs in fibromatosis nodules may explain the high recurrence rate of fibromatosis after excision. One possible explanation is that niches generated by tumor excision stimulate proliferation of stem cells [35]. Although myofibroblasts exist in normal and pathological tissues, the cellular origin of fibromatosis tissues remains unclear. Local mesenchymal cells [36] undergoing epithelial-to-mesenchymal transformation (EMT) [37] are thought to be the cellular origin of pathological myofibroblasts. Future studies are required to determine the relationship between EMT and the involvement of FSCs in fibromatosis nodules. Applications of this model include interventions in the clinical disease course, understanding the signaling or molecular pathways responsible for fibromatosis development, and identifying new therapeutic strategies for the treatment of fibromatosis.

Our study showed that TGF-β1 can induce myofibroblast differentiation of FSCs. Similar effects of TGF-β1 were reported in myofibroblast development or activation in hepatic stellate cells [38], mammary fibroblasts [39] or endothelial cells, renal tubular epithelial cells [40], and neural crest stem cells [41]. A higher level of TGF-β1 has been found in palmar fibromatosis [42] and penile fibromatosis [43] compared with normal tissues. Because TGF-β1 is released after local trauma or after surgical excision [44], the prevalence or recurrence of fibromatosis is associated with previous trauma [45] and surgery history [2]; [13]–[15]. However, the detailed mechanism by which the increase in endogenous TGF-β1 production is involved in the development of fibromatosis requires further investigation. Our study showing a response of FSCs to TGF-β1 in vitro suggests that TGF-β1 plays a role in the development and recurrence of fibromatosis.

FSCs from residual fibromatosis tissues may be stimulated to induce myofibroblast differentiation and collagen deposition, leading to the recurrence of fibromatosis. Given that radical excision of fibromatosis is currently impossible, additional strategies that target FSCs are needed to prevent progression or recurrence after excision. Our study showed that TSA inhibited the proliferation and differentiation of FSCs in vitro and that FSCs pretreated in vivo with TSA did not undergo myofibroblast differentiation and the Matrigel decomposed. Inhibition of myofibroblast differentiation decreased type I collagen accumulation. These data suggest that TSA is a potential adjuvant treatment for fibromatosis that may inhibit the progression or prevent recurrence.

In terms of the clinical applications of TSA, preclinical investigations by several groups have reported the potential efficacy of TSA in the treatment of liver fibrosis [23], breast cancer [46], and squamous cell carcinoma [47]. TSA can inhibit histone deacetylase at nanomolar concentrations, and the resulting histone hyperacetylation leads to chromatin relaxation, causes cell growth arrest [48], and inhibits proliferation [49]. TSA also has been reported to inhibit gene expression of α-SMA and types III and I collagen by inhibiting Sp1 [50]; [51]. Thus, TSA inhibited the proliferation and myofibroblastic differentiation of FSCs, and our results are consistent with this inhibitory ability. Our results suggest that TSA may be useful when given as a local injection into fibromatosis nodules before their progression to cords. Our observation that TSA blocked the formation of fibromatosis nodules by FSCs in vivo suggests that this in vivo murine model may serve in preclinical trials for drug selection to treat fibromatosis.

There remain some limitations to the use of this model that require further clarification. For example, the fibromatosis nodules in the model were created subcutaneously as opposed to the progression of human fibromatosis nodules to fibromatosis cords, which occurs only in specific areas such as the plantar or palmar fascia. The cells used in this study were isolated from palmar fibromatosis, a type of superficial fibromatosis, and future studies are needed with models created by FSCs derived from deep fibromatosis.

### Conclusion

We have successfully isolated and identified the stem cells involved in palmar fibromatosis. Our in vitro and in vivo studies showed that FSCs can undergo myofibroblast differentiation with time to undergo an involutive process involving type I collagen accumulation. The application of FSCs may provide a promising murine model to study the biological and pathological processes responsible for development of fibromatosis nodules, which may lead to therapeutic interventions for fibromatosis. For example, TSA may be a potential therapy for fibromatosis. Understanding the cellular origin of fibromatosis will also enable us to examine the mechanisms underlying myofibroblast differentiation and to delineate targets for adjuvant therapy.

## Supporting Information

Figure S1 **Nodule and cord of human palmar fibromatosis.** (A) H&E staining and immunofluorescence analysis of α-SMA, types III and I collagen. Bars  = 50 µm. (B) The percentages of stained areas. Data are shown as mean ± SD (n = 3). Statistical significance is presented as **, p<0.01 compared with other groups. All experiments were repeated from three different donors.
(TIF)

Figure S2 PCR for genomic DNA contamination in FSCs and 293T cells (A) after 14 days of hepatic differentiation (HIM), (B) after 14 days of neuroglial differentiation (NIM), (C) after 7 days of adipogenic differentiation (AIM), (D) after 7 days of osteogenic differentiation (OIM), and (E) after 7 days of chondrogenic differentiation (CIM). All experiments were repeated with FSCs from three different donors. All experiments were performed with FSCs at passage of 5–8. (Con: without induction).
(TIF)
