# Supplementary figures and images for: Recapitulation of Fibromatosis Nodule by Multipotential Stem Cells in Immunodeficient Mice
Source: PLoS One. 2011 Aug 25;6(8):e24050. doi: 10.1371/journal.pone.0024050 (PMC3162023; doi:10.1371/journal.pone.0024050)

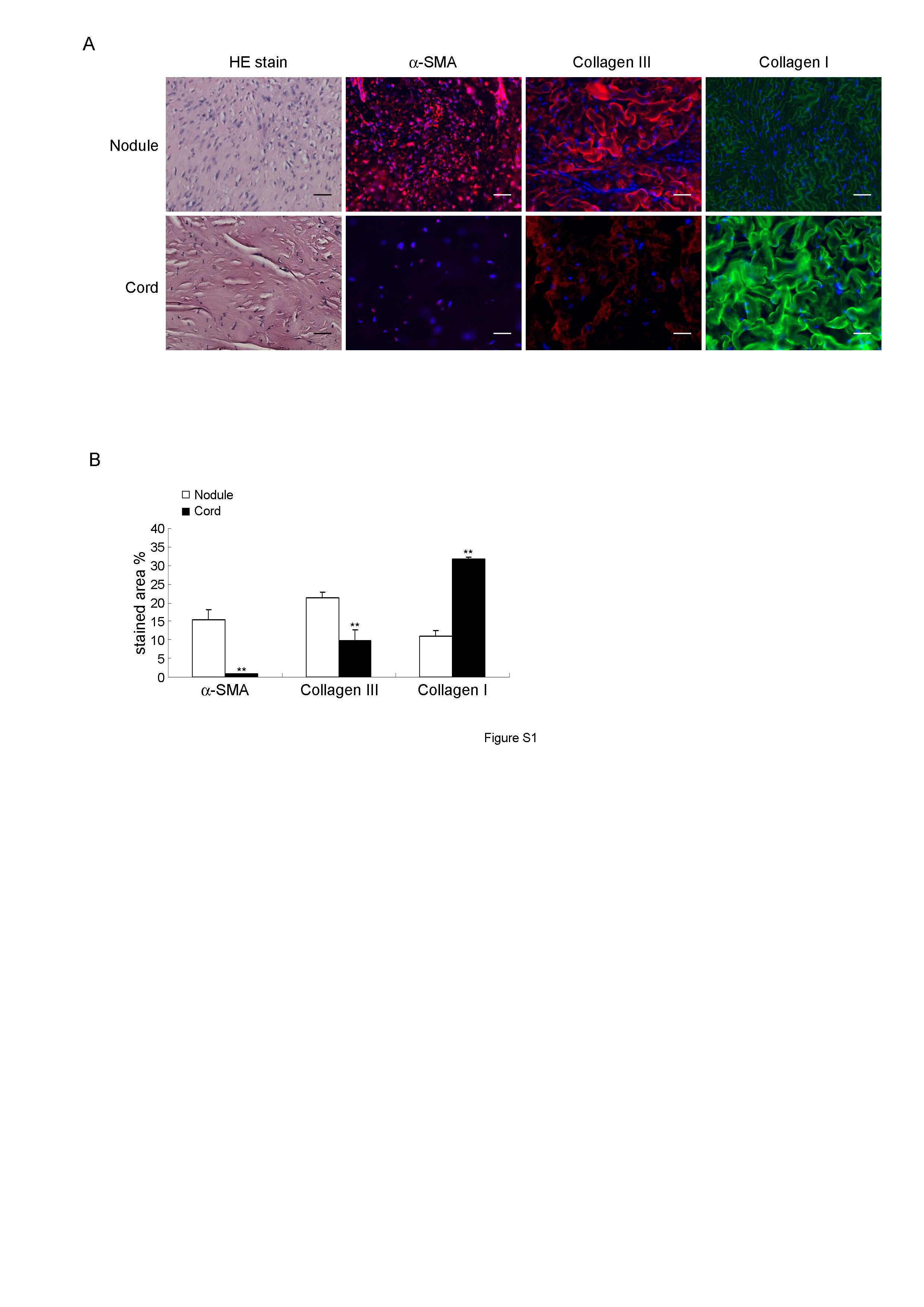

Supplement: Figure S1 — Nodule and cord of human palmar fibromatosis. (A) H&E staining and immunofluorescence analysis of α-SMA, types III and I collagen. Bars = 50 µm. (B) The percentages of stained areas. Data are shown as mean ± SD (n = 3). Statistical significance is presented as **, p<0.01 compared with other groups. All experiments were repeated from three different donors. (TIF) [file pone.0024050.s001.tif]

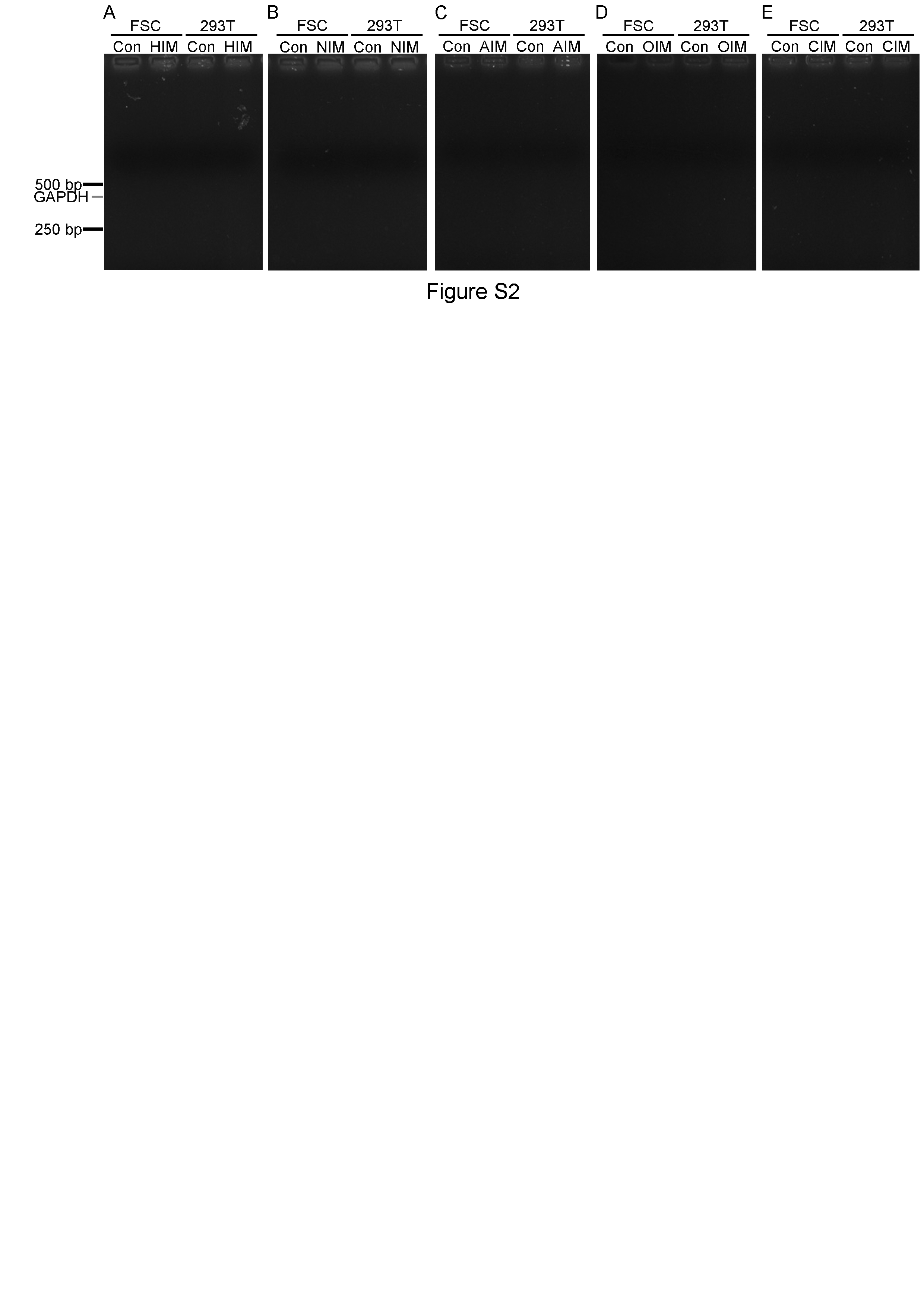

Supplement: Figure S2 — PCR for genomic DNA contamination in FSCs and 293T cells (A) after 14 days of hepatic differentiation (HIM), (B) after 14 days of neuroglial differentiation (NIM), (C) after 7 days of adipogenic differentiation (AIM), (D) after 7 days of osteogenic differentiation (OIM), and (E) after 7 days of chondrogenic differentiation (CIM). All experiments were repeated with FSCs from three different donors. All experiments were performed with FSCs at passage of 5–8. (Con: without induction). (TIF) [file pone.0024050.s002.tif]
